# Comment on: Screening for neuropsychomotor and social-emotional development in children under 24 months of age in the Brazilian semi-arid region

**DOI:** 10.1590/1984-0462/2023/41/2021358

**Published:** 2022-09-09

**Authors:** Karoline Machado Vieira, Maria Eduarda Jeronimo de Oliveira, Eliane Mazzuco dos Santos

**Affiliations:** a Universidade do Sul de Santa Catarina, Tubarão, SC, Brazil.

Dear editors,

The study by Souza et al. highlighted the need for a better assessment of neuropsychomotor and socio-emotional development in children by means of an analysis of sociodemographic and socioeconomic factors and of the Survey of Wellbeing of Young Children (SWYC).^1^ The results pointed to children at risk of developmental delay, especially in the socio-emotional domain, which emphasizes the importance of a screening focused on child development in primary care level to offer adequate care to patients showing early changes.^1^

In addition, when it comes to the family environment, most children had good conditions for development, except for the absence of reading practice. Thus, the objective of this letter is to address the habit of reading as an important aspect in children’s neuropsychomotor and socio-emotional development.

As the study reported changes in 42.2% of the participants when investigating the socio-emotional matter, the habit of reading for children could be an ally to reduce this index, since reading out from birth, when encouraged in primary care, helps to improve emotional development^2^ and to strengthen the affective bond between parents and children, stimulating brain skills and increasing the perception of empathy.^2,3^

It is known that reading is a complex process that requires brain circuits to adapt, and, consequently, it is capable of stimulating neuropsychomotor development.^4^ Therefore, this habit could bring benefits to the development of the child and be used as an ally in proper development of language, increasing children’s vocabulary and boosting concentration.^3,4^ That is, reading can result in clinically important differences in long-term education, given the role of this habit in learning, behavior and cognitive development of a child.^2,3^

That said, it is clear that the screening and evaluation of child development are extremely relevant, as they interfere with the quality of life and social insertion capacity both in childhood and in adulthood.^1,5^ However, this process are of complex interpretation, as it depends on a number of factors. Therefore, the implementation of campaigns aimed at families should be considered, in order to further strengthen socio-emotional training, ensuring that children have access to the means required to develop globally. In this regard, encouraging reading from early childhood is relevant and a possible ally to reduce delays in development, leading a child to grow and develop entirely healthy.
